# Perioperative Temperature Monitoring in Anesthesia: A Review of Current Evidence and Clinical Practice

**DOI:** 10.7759/cureus.107097

**Published:** 2026-04-15

**Authors:** Jason Jogie, Joshua A Jogie, Kenisha Phillip

**Affiliations:** 1 Intensive Care Unit, Port of Spain General Hospital, Port of Spain, TTO; 2 Occupational Health Unit, St. James Medical Complex, Port of Spain, TTO

**Keywords:** anesthesia, core temperature, inadvertent perioperative hypothermia, patient safety, perioperative temperature monitoring

## Abstract

Perioperative hypothermia remains common during anesthesia and surgery. It is associated with higher rates of surgical site infection, blood loss, transfusion, cardiac events, postanesthetic shivering, delayed recovery, and longer hospital stay. Temperature monitoring is therefore an important part of safe anesthesia care when it is linked to thermal management. This article is a narrative review of perioperative temperature monitoring in anesthesia. The aim was to review the physiology of perioperative heat loss, the clinical effects of hypothermia, the performance of common monitoring sites and devices, and the practical role of monitoring in prevention and management. A structured literature review was performed using PubMed. Search terms included combinations of “perioperative temperature monitoring,” “anesthesia,” “hypothermia,” “core temperature,” “esophageal temperature,” “nasopharyngeal temperature,” “bladder temperature,” “zero-heat-flux,” “warming,” and “prewarming.” Boolean operators were used to refine the search. English-language articles relevant to adult perioperative anesthesia care were reviewed. The final manuscript included 43 references, comprising randomized trials, observational studies, reviews, meta-analyses, and guideline-based articles. The evidence shows that core temperature monitoring is more reliable than peripheral or skin-based methods for detecting perioperative hypothermia. Esophageal and nasopharyngeal probes remain practical and accurate during general anesthesia. Bladder temperature is useful in selected patients, especially during longer procedures, but may be less reliable when urine flow is low. Newer zero-heat-flux forehead systems offer a useful noninvasive option across different perioperative phases, although agreement with invasive core sites is good rather than perfect. Evidence also shows that measured temperature can support timely warming or adjustment of warming, better maintenance of normothermia, and lower rates of temperature-related complications. Perioperative temperature should be measured routinely in patients at risk and interpreted together with active thermal management. The method should be chosen according to the patient, the type of anesthesia, and the stage of care. Monitoring is most useful when started early and paired with active warming and clear institutional protocols. More high-quality studies are still needed to compare newer devices, define acceptable accuracy thresholds in different settings, and determine the best monitoring pathway from the preoperative area to recovery.

## Introduction and background

Perioperative temperature monitoring is a basic part of safe anesthesia care, but consistent measurement and action remain variable in practice. In the operating room, even a small fall in core temperature can affect physiology, pharmacokinetics, pharmacodynamics, drug metabolism, coagulation, wound healing, and recovery [1-4]. In this review, inadvertent perioperative hypothermia refers to a core temperature below 36°C, which is a commonly used perioperative threshold [2-4]. It is common during both general and neuraxial anesthesia, and it can begin soon after induction. Temperature monitoring matters because hypothermia may be unrecognized until it is already present, and its effects can be clinically important [1-4].

Normal core temperature is tightly regulated [1,2]. In an awake person, the interthreshold range, which is the narrow temperature range between the onset of sweating and the onset of vasoconstriction or shivering, is small [1,2]. Autonomic responses such as vasoconstriction and shivering are triggered by small changes in core temperature [1]. Anesthetic agents disrupt this control by lowering the vasoconstriction and shivering thresholds and by reducing thermoregulatory behavioral responses [1,2]. General anesthesia also reduces metabolic heat production in many patients and suppresses normal defenses against heat loss [1,5]. This allows heat to move from the warm central compartment to cooler peripheral tissues along the normal core-to-peripheral temperature gradient [1,5]. As a result, core temperature often falls rapidly during the first hour after induction [1,5]. Sessler described this as a major feature of peri-anesthetic heat balance [1], and Matsukawa et al. showed that redistribution accounts for most of the initial decrease in core temperature after induction of general anesthesia [5].

This early fall in temperature is not caused only by heat loss to the room. Redistribution is the main mechanism at first because internal heat shifts from the core to peripheral tissues after anesthetic-induced vasodilation, even before large net environmental heat loss has occurred [1,5]. Later, ongoing cutaneous and respiratory heat loss, open body cavities, cool ambient temperature, cold skin preparation solutions, unwarmed intravenous fluids, and long procedures contribute further [2,6,7]. The typical intraoperative temperature pattern has three phases: a rapid early decline from redistribution, a slower linear decline from net heat loss, and a later plateau when heat loss and production balance or when vasoconstriction returns [2,6,7].

Regional anesthesia also impairs thermoregulation [1,2,8]. Spinal and epidural anesthesia block vasoconstriction in the anesthetized region and alter thermal perception [1,2]. Patients may feel warm even while core temperature is falling [2,8]. Matsukawa et al. showed that redistribution is also a major cause of hypothermia during epidural anesthesia [8]. The decrease in core temperature is often smaller than with general anesthesia because consciousness is preserved, metabolic heat production is less depressed, and unanesthetized regions such as the arms may still vasoconstrict and help limit further redistribution [1,2,8]. Even so, major surgery under neuraxial anesthesia can still lead to clinically relevant hypothermia and should not be viewed as low risk from a temperature standpoint [1,2,8].

The clinical effects of mild perioperative hypothermia are important. One of the strongest early trials was by Kurz et al., who showed that maintenance of normothermia reduced surgical wound infection and shortened hospital stay in patients undergoing colorectal surgery [9]. Frank et al. later showed that perioperative normothermia reduced morbid cardiac events in high-risk patients undergoing major noncardiac surgery [10]. These data helped move perioperative temperature management from a technical issue to a patient safety issue [9,10].

Hypothermia also affects coagulation and blood loss. Schmied et al. reported that mild hypothermia increased blood loss and transfusion requirement during total hip arthroplasty [11]. Even a modest decrease in core temperature impaired hemostasis enough to produce clinically meaningful differences [11]. Hypothermia also promotes postanesthetic shivering, which increases oxygen demand and causes distress in the recovery unit [12]. Alfonsi reviewed postanesthetic shivering and noted that perioperative hypothermia is its main cause, although pain, surgical stress, transfusion, and non-thermoregulatory drug effects may contribute in some cases [12].

Drug effects and recovery are also altered by hypothermia. Lower body temperature slows drug metabolism and may increase anesthetic potency [6,13]. Lenhardt et al. showed that mild intraoperative hypothermia prolonged postanesthetic recovery and delayed readiness for discharge from the postanesthesia care unit [13]. This effect is thought to occur because hypothermia slows hepatic and renal drug clearance, alters tissue uptake and release of anesthetic agents, and delays recovery from anesthesia [6,13]. These effects matter in modern perioperative pathways where rapid recovery, early mobilization, and efficient operating room flow are expected [6,13]. Hypothermia also increases discomfort, and patients often remember feeling cold as a negative part of their perioperative experience [6].

Many of these complications are reduced when perioperative hypothermia is prevented, so temperature should be measured rather than guessed [2-4,9-14]. Reviews by Sessler, Insler and Sessler, and Forstot emphasized that temperature is an important perioperative vital sign and a routine part of anesthesia assessment [2-4]. Sessler stated that body temperature should be measured in patients undergoing general anesthesia lasting more than 30 minutes and in patients having major surgery under neuraxial anesthesia [2]. Torossian et al., in a clinical practice guideline, recommended preoperative measurement and then continuous or at least regular intraoperative measurement, because prevention depends on early recognition [14].

The key question is what temperature should be monitored and where. Core temperature is the target because it reflects the temperature of vital organs and informs assessment of central thermoregulatory status under hypothalamic control [1-4]. Reliable core sites include the pulmonary artery, distal esophagus, nasopharynx, bladder, rectum, and direct tympanic membrane measurements [2-4,15]. The pulmonary artery is the reference standard in critically ill patients, but is invasive and not used for routine anesthesia [2,3]. In everyday anesthesia practice, the esophagus and nasopharynx are widely used under general anesthesia because they provide continuous readings with good agreement with core temperature [2-4,15]. Rectal temperature reflects core temperature reasonably well in stable states but responds more slowly during rapid temperature change [2,7,15]. Cork et al. showed that nasopharyngeal, esophageal, and bladder measurements were more precise than axillary, forehead, and toe measurements [15].

Noninvasive methods remain attractive, especially before induction, during regional anesthesia, and in the recovery room. But they have limitations. Sessler noted that no site that is both noninvasive and easy to use is reliably core in all patients [2]. Oral temperature is among the better noninvasive options in cooperative patients [2,4,16], while infrared ear thermometry is less reliable unless the tympanic membrane itself is measured directly [2,16]. Torossian also concluded that oral measurement is the most reliable noninvasive route and that infrared ear methods are inaccurate for perioperative decision-making [16]. Skin temperature alone should not be used as a surrogate for core temperature during anesthesia [2,4,16].

Newer technologies try to address this limitation. Zero-heat-flux forehead systems allow continuous noninvasive monitoring and are increasingly used in operating rooms and recovery areas. In a systematic review and meta-analysis, Conway et al. found a small pooled mean bias compared with core temperature, but the limits of agreement were broad enough that clinicians should recognize uncertainty of up to about 1°C in either direction [17]. Morettini et al. reported clinically acceptable agreement between zero-heat-flux and esophageal temperature during major surgery, with most paired values within ±0.5°C [18]. These data suggest that newer noninvasive systems can be useful, but performance depends on the clinical setting and the level of precision required [17,18].

Temperature monitoring should also be linked to prevention. Monitoring alone does not maintain normothermia, but it can prompt timely intervention. Prewarming before induction can reduce redistribution hypothermia by increasing peripheral heat content [4,14,19]. Forstot highlighted preinduction skin-surface warming as the only way to blunt the initial redistribution drop [4]. Torossian et al. recommended active prewarming for 20 to 30 minutes when feasible [14]. Grote et al. later showed lower rates of intraoperative and postoperative hypothermia in patients who underwent prewarming according to guideline-based practice [19].

Active intraoperative warming is also well supported. Forced-air warming is the most commonly used noninvasive method and remains highly effective [20-22]. Meta-analyses have shown that forced-air warming prevents perioperative hypothermia better than passive insulation and some older warming methods, though it may be similar to other active systems in some settings [20-22]. Heating intravenous fluids is also important when large volumes are given [3,7]. Insler and Sessler noted that warmed fluids do not warm a patient by themselves, but they do prevent additional cooling caused by cold infusions [3]. The overall principle is simple: measure temperature early, track it when risk is present, and intervene before hypothermia becomes established.

Despite strong evidence, practice remains variable. Bindu et al. noted that temperature continues to be one of the least seriously monitored perioperative variables [23]. Surveys have shown gaps in consistent monitoring and warming, even though clinicians recognize the problem [3,23]. This practice variation may reflect limited access to equipment, short cases being viewed as low risk, uncertainty about which site to use, or underestimation of the effect of mild hypothermia [3,23].

Materials and methods

This article was written as a narrative review. It was not designed or reported as a formal systematic review, and no study-selection flow diagram or pooled meta-analysis was used. The purpose was to provide a clinically focused summary of perioperative temperature monitoring in anesthesia using a structured review of the literature.

A structured search of PubMed was performed to identify relevant articles. Because the reference list for this manuscript was limited to PubMed-indexed literature, no additional database was used for article selection. The search was focused on studies and reviews relevant to adult perioperative anesthesia care. Search terms included combinations of the following keywords: “perioperative temperature monitoring,” “temperature monitoring,” “perioperative hypothermia,” “inadvertent perioperative hypothermia,” “anesthesia,” “general anesthesia,” “neuraxial anesthesia,” “core temperature,” “esophageal temperature,” “nasopharyngeal temperature,” “bladder temperature,” “rectal temperature,” “zero-heat-flux,” “prewarming,” “forced-air warming,” and “warming systems.” Boolean operators such as AND and OR were used to refine results. Example search strings included “perioperative hypothermia AND anesthesia,” “core temperature AND esophageal OR nasopharyngeal,” “zero-heat-flux AND perioperative temperature,” and “prewarming AND anesthesia AND hypothermia.”

The literature reviewed spanned studies published from 1983 to 2024. Articles were selected for clinical relevance, quality of evidence, and direct relation to the topic. Included article types were randomized controlled trials, observational studies, systematic reviews, meta-analyses, guideline-based reviews, and major narrative reviews relevant to perioperative temperature monitoring and thermal management. Preference was given to studies that addressed one or more of the following areas: thermoregulatory physiology during anesthesia, clinical consequences of hypothermia, accuracy of temperature-monitoring sites, performance of newer monitoring devices, risk factors for hypothermia, and the effect of warming strategies on measured temperature and clinical outcomes.

Articles were excluded if they were not indexed in PubMed, were not clearly related to perioperative temperature monitoring, focused mainly on non-perioperative settings, or did not add meaningful data to the clinical narrative of the review. Non-English articles were not intentionally sought. The final manuscript included 43 references.

No formal quantitative synthesis was performed. No statistical software was used because there was no pooled analysis, meta-analysis, or original dataset. Data were reviewed and synthesized narratively. No formal scoring tool or scale was applied within this review, and no copyrighted clinical score was used as an outcome measure. The main aim was practical interpretation of the literature for clinicians involved in perioperative anesthesia care.

## Review

Accuracy of monitoring methods

Perioperative temperature monitoring should be treated as routine care that informs thermal management, not as an isolated extra step. Hypothermia is common during anesthesia, and it is often missed unless core temperature is measured in a reliable way. Monitoring helps identify patients at risk, assess whether a device reflects core temperature well enough for clinical use, and guide warming before, during, and after surgery.

The temperature monitor should be accurate, noninvasive, continuous, inexpensive, and easy to use before induction, during surgery, and in recovery. No single method meets all of these goals. In practice, clinicians choose between invasive or semi-invasive core sites and less invasive surrogate sites. The problem is that ease of use often comes at the cost of lower precision.

**Figure 1:**
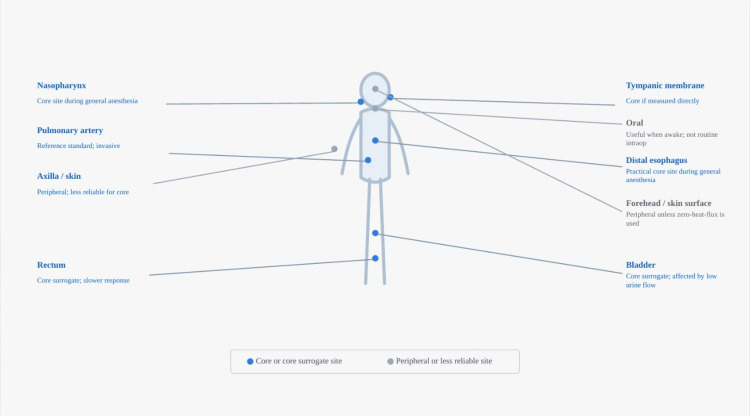
Common perioperative temperature monitoring sites Common perioperative temperature monitoring sites. Blue labels mark core or core surrogate sites commonly used in anesthesia. Gray labels mark peripheral or less reliable sites. Figure created in Microsoft PowerPoint (Microsoft Corporation, Redmond, WA, USA) using native shapes and line work.

Figure 1 shows the common perioperative temperature monitoring sites used in anesthesia and highlights core and peripheral locations discussed in this review.

Under general anesthesia, the esophagus remains one of the most practical reference sites because it is easy to access in intubated patients, provides continuous values, and tracks core temperature well [15]. The nasopharynx is also widely used, but accuracy depends on probe position [15]. Iden et al. compared zero-heat-flux technology with sublingual and nasopharyngeal measurements during general anesthesia and showed close agreement with nasopharyngeal values [24]. Eshraghi et al. evaluated a zero-heat-flux cutaneous thermometer in cardiac surgical patients and found a small bias compared with pulmonary artery temperature, though precision was wider than preferred [25]. West et al. reported moderate agreement between zero-heat-flux and naso-/oropharyngeal probes during anesthesia [26]. Munday et al. found similar results when zero-heat-flux monitoring was compared with esophageal temperature in orthopedic surgery [27]. Munday et al. also showed that implementation of continuous temperature monitoring improved perioperative monitoring practice, especially in the recovery area [28]. Liang et al. later reported agreement of zero-heat-flux thermometry with oesophageal and tympanic core temperature measurement in patients receiving major surgery [29].

Bladder temperature is useful when urinary catheterization is already planned. Bone & Feneck found that bladder temperature performed reasonably well compared with esophageal, nasopharyngeal, and rectal temperature in cardiac surgical patients [30]. Lefrant et al. later showed in critically ill adults that urinary bladder and esophageal measurements were more reliable than rectal thermometry [31]. Erdling & Johansson compared esophageal and nasopharyngeal temperature in the same anesthetized patients and found that the two methods were close, but not identical [32]. Lim et al. showed that temperatures differed across commonly used nasopharyngeal positions, which means depth and placement matter [33]. Taken together, these studies show that esophageal and nasopharyngeal monitoring remain dependable during general anesthesia, while bladder monitoring is useful in selected patients but may lag during rapid thermal change [30-33].

Rectal and skin measurements are less attractive when accurate real-time core data are needed. Rectal temperature changes more slowly and may not detect rapid intraoperative drops early enough [2-4,16,31]. Skin temperature is even less dependable because it is strongly affected by ambient temperature, perfusion, and local warming devices [2,15,16,31]. These weaknesses matter because prevention depends on early detection. A monitor that lags behind true core change may delay intervention long enough for hypothermia to become established.

Table 1 summarizes the main monitoring methods, including accuracy, lag time, and clinical feasibility.

**Table 1:** Practical comparison of common perioperative temperature-monitoring methods

Monitoring site/device	Core or peripheral	Accuracy for core temperature	Response lag	Clinical feasibility	Main limitations
Distal esophagus	Core	High [2-4,15,32]	Low	High in intubated patients	Not available in awake or non-intubated patients
Nasopharynx	Core	High when correctly positioned [15,32,33]	Low	High during general anesthesia	Accuracy depends on placement depth
Bladder	Core surrogate	Moderate to high [30,31]	Moderate	Useful when catheter already in place	Less reliable with low urine output
Rectum	Core surrogate	Moderate [2-4,16,31]	Higher	Easy in selected cases	Slower response during rapid change
Oral	Peripheral/core surrogate	Moderate in awake cooperative patients [2,4,16]	Moderate	Preoperative and postoperative use	Not suitable during most intraoperative care
Skin/axillary/forehead without zero-heat-flux	Peripheral	Low for core trends [2,15,16,31]	Variable	Easy	Strongly affected by environment and perfusion
Zero-heat-flux forehead system	Core surrogate	Moderate to high [17,18,24-29]	Low to moderate	Very useful across phases of care	Agreement is good, but not identical to invasive core sites

Risk factors for perioperative hypothermia

Monitoring is most useful when linked to risk. Observational studies have identified several predictors of intraoperative hypothermia. Sari et al. found high rates of perioperative hypothermia and identified longer anesthesia and surgery duration, advanced age, higher ASA class, major surgery, endoscopic procedures, overweight, and unwarmed fluid administration as important risk factors [34]. Vural et al. also identified large or moderate surgical magnitude, preoperative temperature below 36°C, combined regional and general anesthesia, age over 70 years, and relevant comorbidity as contributors [35]. Yi et al., in a national study in China, found that active warming, higher baseline temperature, higher ambient temperature, and higher body mass index reduced risk, while major-plus surgery and anesthesia duration over two hours increased risk [36]. These findings are clinically useful because they help decide when continuous core monitoring is most important.

These risk factors also explain why short cases should not always be viewed as low risk. A patient who starts cool, receives a neuraxial block, has unwarmed fluids, or is cared for in a cool room can become hypothermic even during a moderate-length procedure. Monitoring should therefore be based on the whole thermal risk profile rather than case length alone.

Prewarming and prevention

Temperature monitoring becomes meaningful when it leads to prevention. One of the most studied strategies is prewarming. The idea is to increase peripheral heat content before induction and reduce the redistribution drop in core temperature. Evidence supports this approach, but the size of the benefit depends on the population and the overall warming protocol.

Andrzejowski et al. showed that prewarming before general anesthesia reduced post-induction temperature decrease and improved maintenance above the 36°C threshold [37]. Lau et al. later found that at least 30 minutes of preoperative forced-air warming reduced intraoperative hypothermic exposure, although redistribution hypothermia was not abolished [38]. Recio-Pérez et al. found no significant reduction in incidence, duration, or magnitude of hypothermia in patients receiving loco-regional or general anesthesia, although this occurred within a strict temperature-management protocol and a relatively low baseline hypothermia rate [39]. Shirozu et al. reported that prewarming during epidural catheter insertion could safely reduce redistribution-related core temperature decrease [40]. Chung et al. showed that preoperative forced-air warming and warmed fluid reduced hypothermia and shivering during elective cesarean delivery under spinal anesthesia [41]. This body of work suggests that prewarming works best when baseline warming practice is otherwise limited or when redistribution risk is high.

These findings do not weaken the case for monitoring. They strengthen it. When clinicians can see temperature in real time, they can judge whether prewarming was enough, whether forced-air warming should begin earlier, or whether another cause of heat loss needs attention.

Intraoperative warming and temperature-guided care

Active body surface warming remains a major part of prevention. Madrid et al., in a Cochrane review, found that active body surface warming systems reduce inadvertent perioperative hypothermia and related complications better than passive insulation alone [42]. Temperature monitoring is needed to make these systems effective, rather than routine but blind. Without measured core temperature, clinicians may under-treat patients who continue to cool or over-treat those who are already normothermic.

Other thermal measures, such as warming intravenous fluids and irrigation solutions, are best viewed as adjuncts. They reduce avoidable heat loss but may not be enough alone in moderate- or high-risk surgery. The strongest approach remains combined care: baseline risk assessment, reliable temperature monitoring, and active warming adjusted to the measured trend.

Outcomes linked to hypothermia and the role of monitoring

Observational outcome studies reinforce why monitoring matters. Yi et al. found that intraoperative hypothermia was associated with more postoperative shivering, higher ICU admission, and longer stay in the postanesthesia care unit and hospital [36]. Lopez reviewed postanesthetic shivering and noted that hypothermia remains the main trigger in many patients, although not the only one [43]. Shivering is not trivial. It increases oxygen use and discomfort and may worsen recovery in patients with limited cardiopulmonary reserve. Since prevention of hypothermia is the first step in preventing shivering, temperature monitoring has direct value after surgery as well as during it.

There is also a process-of-care argument. A monitor changes behavior. It prompts earlier warming, identifies device failure, and confirms whether the patient has rewarmed before discharge from recovery. This is especially important in settings where temperature has traditionally been checked less often than blood pressure or oxygen saturation.

Practical implications

The evidence supports a practical hierarchy. During general anesthesia, distal esophageal and well-positioned nasopharyngeal probes remain the most dependable routine monitors. Bladder temperature is useful in catheterized patients, especially during long cases. During neuraxial anesthesia, before induction, during transfer, and in the recovery unit, zero-heat-flux systems offer a strong noninvasive option, though agreement with core reference methods is good rather than perfect. Oral temperature may help in selected awake patients, but skin and superficial peripheral readings should not guide important intraoperative decisions when better options exist.

The main unresolved issues are now practical rather than conceptual. Which monitor is accurate enough for each phase of care? How much precision is needed to trigger warming? And how can monitoring be integrated into busy perioperative workflows so it changes outcomes rather than just creating more data? Current evidence suggests that continuous monitoring across the perioperative pathway is feasible and useful, especially when paired with active warming and clear protocols. But device selection should still be based on the clinical context, not convenience alone.

Limitations

This review has limitations. First, it is a narrative review and not a formal systematic review. The literature was reviewed in a structured way, but no formal study-selection flow diagram, risk-of-bias tool, or pooled meta-analysis was used. Second, only PubMed-indexed references were included, which may have excluded relevant studies indexed elsewhere. Third, the review includes different study types, including randomized trials, observational studies, reviews, and meta-analyses. This broad approach is useful for clinical discussion, but it also means the evidence is not uniform in design or strength. Fourth, several monitoring methods perform differently across surgical populations, levels of warming, and phases of care, so direct comparison between studies should be interpreted with caution. Fifth, newer noninvasive technologies continue to evolve, and future device updates may change performance relative to older published studies.

Future directions

Future work should focus on clinically useful questions. More high-quality comparative studies are needed between established core sites and newer noninvasive systems, especially in neuraxial anesthesia, ambulatory surgery, and postoperative recovery. Standard definitions of acceptable bias and limits of agreement would also help clinicians judge whether two methods are interchangeable in practice. Research should also examine full perioperative monitoring pathways rather than isolated intraoperative measurements, because hypothermia often begins before induction and may persist after surgery. In addition, future studies should link monitoring strategies to patient-centered outcomes such as shivering, recovery time, unplanned ICU admission, and hospital length of stay. Finally, more implementation studies are needed to understand how protocols, staffing, and equipment access affect whether temperature monitoring is used consistently in routine anesthesia care.

## Conclusions

Perioperative temperature monitoring is a basic part of safe anesthesia care. Inadvertent hypothermia remains common and can lead to worse outcomes, including more bleeding, wound infection, cardiac stress, shivering, delayed recovery, and longer hospital stay. Reliable core temperature monitoring helps detect early temperature drop and supports timely warming. Esophageal and nasopharyngeal probes remain practical and accurate during general anesthesia, while newer zero-heat-flux devices may be useful when noninvasive continuous monitoring is needed. No single method is best in every setting, so the choice should depend on the patient, the type of anesthesia, and the stage of care. Temperature monitoring should begin early and continue through the perioperative period in patients at risk. It should also be paired with active warming and clear protocols. More high-quality studies are still needed to compare newer devices and to define the best monitoring strategy in different surgical settings.
